# ‘All I Do Is Sit in a Chair Until the Pain Fades’—Experiences of Living With Gout

**DOI:** 10.1111/hex.70302

**Published:** 2025-05-21

**Authors:** Helene Sedelius, Renée Flacking, Mats Dehlin, Anna Svärd, Malin Tistad

**Affiliations:** ^1^ School of Health and Welfare Dalarna University Falun Sweden; ^2^ Center for Clinical Research (CKF) Dalarna Uppsala University Falun Sweden; ^3^ Department of Rheumatology and Inflammation Research, Institute of Medicine, Sahlgrenska Academy University of Gothenburg Gothenburg Sweden; ^4^ Department of Biomedical and Clinical Sciences Linköping University Linköping Sweden; ^5^ Department of Neurobiology Care Sciences and Society Karolinska Institutet Stockholm Sweden

**Keywords:** gout, grounded theory, lived experience, patient, primary care

## Abstract

**Background:**

Living with gout impacts most dimensions of life. However, there is a lack of studies exploring the trajectory of patients' experiences of living with and being treated for gout, beyond the experiences during a flare. This study aimed to explore how individuals with gout experience the disease, its effects on daily life and their encounters with healthcare.

**Methods:**

A constructivist grounded theory was used, involving simultaneous data collection and analysis. Semi‐structured individual interviews were conducted with 12 individuals living with gout and aged between 40 and 87 in Central Sweden.

**Results:**

Navigating the uncertainty of living with gout was represented through two categories: ‘a mismatch between individuals' needs and the provision of care’ and ‘a process of adaptation’. The mismatch involved unmet needs for pain relief, feeling dismissed as having a minor condition and a lack of personalised care. The adaptation process included seeking explanations, developing self‐management strategies and adjusting to pain and functional limitations.

**Conclusions:**

Living with gout entails a significant degree of uncertainty. The process of adaptation is affected by a mismatch between individuals' needs and the care provided, in addition to the disease's ‘roller coaster’ nature and its slow progression.

**Patient and Public Contribution:**

This study is part of a research project aimed at gathering knowledge essential for developing an intervention in primary care. A patient, appointed by the Swedish Rheumatism Association, is actively participating in the project's research group. The results of this study have been discussed and analysed within the research group, including input from the patient participant.

**Abbreviations**
p: participants
n: number of participants
ULT: urate‐lowering treatment

## 1 Background

Living with gout and experiencing gout flares has a multifaceted impact on most dimensions of life, including physical functioning, daily activities and psychological and social well‐being [1, 2]. For some individuals, living with gout entails enduring pain and reduced mobility, as well as worrying about gout flares [1, 3].

Gout is a common condition, and its global prevalence has increased over the last three decades, affecting around 2% of the population [4, 5, 6], men more often than women. Women are older (67 years) at onset compared to men (56 years). Women are more obese with higher use of diuretics [7]. It is associated with severe pain, joint destruction and, for some individuals, the development of a chronic condition over several years due to repeated gout flares [8]. Moreover, gout is associated with increased absenteeism from work [9] and a higher risk of mortality [10]. Prophylactic urate‐lowering treatment (ULT) has been available for a long time. It is effective [11, 12], inexpensive, has minimal side effects [13] and offers a possible cure for gout [14]. Even though Swedish and European recommendations advocate the use of prophylactic ULT [15, 16], treatment is often limited to pain relief. National and international studies report that only one‐third of patients with gout receive ULT [4, 5, 17, 18], and two‐thirds experience insufficient control of their gout [19]. This low proportion receiving adequate treatment leads to preventable healthcare visits and hospital admissions [20, 21]. Moreover, less than half of patients prescribed ULT continue their treatment over time [22]. According to the Swedish treatment recommendations, ULT should be prescribed, often lifelong, after a gout flare in patients with at least one other risk factor (e.g., more than one joint affected or the presence of comorbidities). The recommendation also emphasises non‐pharmacological advice about reduced alcohol consumption and diet adjustments, weight loss, increased physical activity and individualised patient education [16]. Several barriers to the provision of ULT have been reported. These include healthcare professionals' inadequate knowledge and misconceptions about gout and discontinuity in healthcare [20, 23, 24, 25], as well as patients' misconceptions about gout, low confidence in general practitioners (GPs) and feelings of shame when seeking care, due to the stigma that gout is caused by poor lifestyle habits and thus self‐inflicted [20, 23].

A common sentiment among gout patients is a sense of resignation to the disease and a loss of belief in the effectiveness of further actions [19]. Pain, together with mobility difficulties [26], anxiety between flares due to uncertainty about when the next flare will occur [1, 3] and irritability that affects family relations [27] are recurring experiences of living with gout.

Hence, to improve the care provided to patients with gout, it is essential to investigate their experiences of living with gout and their interactions with healthcare over time. As indicated by Garcia‐Guillen et al. [2], there is a lack of long‐term studies exploring patients' perspectives across the four identified domains: social life, psychological health, physical functioning and family life, beyond the short period of a gout flare. Furthermore, we did not identify any comprehensive studies that explore the trajectory of patients' experiences of living with and being treated for gout, extending beyond the typical flare duration (1–4 weeks). Therefore, the aim of this study was to explore how individuals with gout experience the disease, its impact on daily life and their encounters with healthcare.

## 2 Methods

A constructivist grounded theory approach was used to explore the experiences of individuals living with gout. Grounded theory utilises a constant comparative method, where data collection and analysis are carried out in parallel to generate theories that explore complex social processes. This approach was chosen to capture rich and detailed descriptions of participants' experiences. Unlike the grounded theory developed by Glaser and Strauss [28], constructivist grounded theory presumes that researchers' pre‐understandings are important in the research process. The presentation of detailed descriptions is created from the interaction between the researcher, previous knowledge in the field and the study participants [29].

### 2.1 Setting

In Sweden, gout is primarily managed in primary care, with specialist care serving as consultants. The healthcare system in Sweden is tax‐funded and covers all legal residents. The country is divided into 21 self‐governing regions, each responsible for providing healthcare to all residents [30]. This study included participants from six primary care units located in three regions in Central Sweden.

### 2.2 Participants

Individuals over age 18 with a diagnosis of gout (ICD‐10 M10) and at least one recorded visit to a primary care unit in the past year were eligible for inclusion. A purposive selection based on age, gender, duration of gout and urban/rural residency was employed. Care administrators, following the routines in each participating region, identified eligible individuals using medical records. Potential participants received a letter with brief information about the study and were contacted by telephone to receive full details. The interview's location, time and mode (telephone/face‐to‐face/digital video meeting) were planned in accordance with the participant's wishes. Before the interview, participants received written and verbal information about the study and signed a consent form.

Twenty‐nine potential participants were contacted by letter, of which some declined participation over the phone, and others could not be reached. Ultimately, 12 individuals agreed to participate: 8 men and 4 women aged between 40 and 87 years, and 3 under 65 years, with a median age of 74. The duration of the disease ranged from 1 to 35 years, totalling 143 years when summed up for all participants. Eight participants had a disease duration of more than 3 years. Four suffered from more than two gout flares per year. Most participants (*n* = 10) were married/cohabiting. Three lived in apartments, and nine resided in privately owned houses. Half of the participants (*n* = 6) lived in urban areas, and the others lived in the countryside. Most participants (*n* = 10) had experience using ULT (allopurinol) at a dose between 100 mg and 300 mg per day. A majority (*n* = 10) also had other morbidities, such as cardiovascular diseases, diabetes, cancer and stroke.

The study was approved by the Swedish Ethical Review Authority (reg number 2019‐00077 and 2019‐04888). Citations have been carefully chosen to ensure that individuals could not be identified, and participants are referred to as participants (p) 1–12.

### 2.3 Data Collection and Analysis

Before data collection began, the first author (H.S.) was interviewed by a co‐author (R.F.) with the purpose of illuminating her pre‐understandings. Shortly thereafter, a pilot interview was conducted to test the interview guide; however, it was not included in the data collection. The first author (H.S.) performed all the interviews between June 2021 and November 2022. Four of the interviews were conducted through digital video meetings, while the remaining interviews were conducted face‐to‐face. The interview guide comprised questions such as: Can you tell me about your experiences of gout? How do you feel about your treatment? What are your experiences with healthcare related to gout? The interviews lasted, on average, 33 min (range 23–47 min). Reflective notes were written directly after the interviews, encompassing both the content of the interviews and the progression of the analysis. These notes were used throughout the simultaneous and iterative process of collecting and analysing the data. The interviews were recorded and transcribed verbatim. The initial coding was performed in a manner that was aligned with the text and was done for all of the interviews, with the purpose of not losing important content. Moreover, a mind map was constructed after coding each interview. After five interviews, the interview guide was supplemented with relevant topics based on the analysis. These supplementing topics were intended to capture a broader and more vivid description of the participants' experiences. During the parallel process of data collection and analysis, variation in participants' experiences was sought, using theoretical sampling to refine categories and subcategories. This was done targeting parts of the analysis that needed to be substantiated when interviewing additional individuals. In addition, towards the end of the process, transcripts were re‐read to identify variations that could support connections between categories. Tentative categories were identified, which were essential for the iterative process of going back and forth between the original text and the abstract level. A tentative core category was constructed after eight interviews and was tested and modified throughout the iterative process. The content of the categories and the links between them were compared and changed several times during the writing process. To ensure that the created categories and conclusions drawn were grounded in the data, continuous discussions were held with co‐authors throughout the parallel processes of recruitment, data collection and analysis. To enhance the trustworthiness of the interpretation, the findings were discussed with a patient representative and research partner based on their own lived experiences of gout. Saturation was considered to be achieved when no new prominent contributions were made to the existing categories or how they were related, that is, no new patterns were to be identified; thus, no further interviews were deemed necessary after the 12th interview.

## 3 Results

### 3.1 Navigating the Uncertainty of Living With Gout

**Figure 1:**
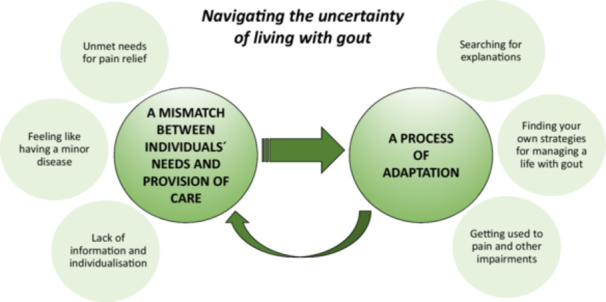
The model visualises categories and subcategories within the context of the core category ‘navigating the uncertainty of living with gout’.

The core category *navigating the uncertainty of living with gout* describes participants' experiences of both gout and the healthcare system and consists of two categories. The core category and the categories and subcategories are presented in Figure 1. The first category, a mismatch between individuals' needs and the provision of care, describes important factors related to healthcare and society that affect the second category: a process of adaptation. Subcategories for the first category include unmet needs for pain relief, the feeling of having a minor disease and a lack of information and individualised care. Three subcategories substantiate the process of adaptation: searching for explanations, finding your own strategies for managing a life with gout and getting used to pain and other impairments.

#### 3.1.1 A Mismatch Between Individuals' Needs and the Provision of Care

There was a mismatch between the needs perceived by participants and the support and treatment they experienced from healthcare services. This mismatch arose because the pain and the disease progression over time were experienced as severe in relation to the almost non‐existent recognition of this experience and actions taken by healthcare personnel to meet the needs.

##### 3.1.1.1 Unmet Needs for Pain Relief

The severity of the pain during a gout flare was described as worse than any pain experienced before. They described their pain as ‘crashing’, ‘unbearable’, ‘troublesome’, ‘dull yet explosive’, ‘sensitive to even a light touch’, ‘burning’ and ‘physically debilitating’. Many expressed a strong desire to rid themselves of the affected body part: ‘It was like … yes, it was like if you have had an axe and could chop off the toe, so to say; that's how painful it was’ (p 5). The pain was perceived as more distressing when undiagnosed.

Every participant vividly recalled their first gout flare, whether it happened 2 or 30 years ago. The intense pain made them expect care that matched its severity. These expectations were not always met. Some participants reported being told during their initial acute visit that the pain would resolve within 1–2 weeks and were sent home, occasionally without pain medication. While some participants experienced no delays in receiving care from their primary care units, others faced challenges. One man was advised over the phone to wait 1 week more before seeking further medical care. As a result, he chose to contact occupational healthcare instead.

It's so hard to get an appointment, and it hurt so much. They said, ‘in a few weeks or in 1 week’. It was a pressured situation for them at the time. They may not be the ones to blame, but it's really hard for me to accept as a person, when you're in such terrible pain.

(p 2)

##### 3.1.1.2 Feeling Like a Person With a Minor Disease

During their initial visits, participants often felt that healthcare personnel perceived gout as an insignificant ailment. They noted that conditions like diabetes or hypertension received greater attention, with care plans established early on. One man with diabetes and gout stated: ‘If I compare with diabetes where I have follow‐up every year … my gout, it's a closed chapter for healthcare’ (p 8).

One participant mentioned that there seemed to be no space within healthcare for gout. The message of ‘just wait and ease the pain’ further reinforced the perception that gout was considered minor. ‘They didn't really listen to me; they told me what it was. I didn't get any preventive treatment, only pain relief … in the end, I got another physician’ (p 8). The diagnostic process felt rushed during their initial acute healthcare visits, which left them unconvinced that they had been diagnosed correctly. All participants reported a lack of consistency in the care provided. Blood sampling and the prescription of preventive treatment seemed to happen in a haphazard manner. One man described that no blood sampling was done, yet: ‘They wanted to triple the dose right away … it didn't feel ok’ (p 2). The experiences from these initial healthcare encounters significantly influenced participants' decisions on when to seek further care for their gout. Those who had a positive first encounter were more likely to seek help earlier for subsequent flares.

In some cases, several years passed before preventive treatment (ULT) was prescribed, while for others, it was done at the first visit. One participant mentioned that he did not receive preventive treatment until he, accidentally, changed physicians. Many participants were unsure if they could expect more from their healthcare providers, leaving them wondering if they were being denied essential gout treatment. One individual narrated: ‘I had received pain‐relieving drugs…. I didn't know if I could have received other or better care’ (p 7).

##### 3.1.1.3 Lack of Information and Individualisation

Participants' experiences with healthcare visits were often characterised by limited time, making it difficult to ask questions and fully comprehend the information provided about gout and its treatment. They described counselling about treatment as inadequate and not tailored to their individual needs. A recurring concern was the perceived inability of healthcare professionals to empathise with them, which often resulted in poor communication. One participant experienced a profound lack of individualised support due to her specific needs following a stroke.

I've tried to get help … like meeting one and the same physician … because I have a bit of a hard time keeping up when they're talking. Having the same physician that has an awareness about my stroke.

(p 12)

Participants perceived a lack of individualised guidance, with healthcare personnel overemphasising general lifestyle advice. One participant expressed this sentiment by stating, ‘It was a lot about what I shouldn't eat’ (p 2). Several participants felt that the advice provided, such as dietary changes, doing more exercise and weight loss, was too generic and not tailored to their needs. This approach did not account for the nature of gout, which is often characterised by pain that interferes with physical activity. The inadequate counselling from healthcare providers was especially disturbing for younger participants, especially those with physically demanding jobs or active lifestyles. One participant found the health‐promoting lifestyle changes recommended by his physician overwhelming and disproportionate compared to other treatments presented. Despite making significant lifestyle changes to address his diabetes and lose weight, no improvement was noticed. Another participant with similar experiences described how it made him question the suggestion that gout was solely a lifestyle‐related disease, as suggested by his physician.

During healthcare visits, due to other diseases, gout was rarely addressed. Participants with prescriptions for ULT and pain‐relieving drugs often contacted healthcare providers by phone or digital messages when they were running out of medication. The participants expressed a longing for continuity in their care, emphasising the importance of seeing the same physician for each visit. This consistency, they believed, would enable a more individualised counselling and improved gout management.

#### 3.1.2 A Process of Adaptation

The process of adaptation involved an emotional and intellectual struggle to comprehend gout, alongside constructive efforts to address its social and physical impacts. At the same time, participants gradually habituated to the pain and physical impairments associated with the disease. These efforts were integral to adapting to a disease that slowly worsened over many years. Additionally, the unpredictable nature of gout, characterised by sudden flares, added to the challenge of accepting and navigating the uncertain terrain of the condition.

##### 3.1.2.1 Searching for Explanations

Participants felt poorly informed about gout and often sought their own explanations, with many lacking understanding of its causes despite years of living with the condition. Some were aware of the involvement of urate crystals, ‘it's about the uric acids in the blood’ (p 6), but their knowledge appeared to be incomplete. Most of the information they had about gout came from conducting online searches. One participant noted, ‘Then I read about it on the internet’ (p 11).

A few participants expressed a preference for not obtaining extensive knowledge about gout, simply desiring proper treatment without delving into its causes or the mechanisms of the medications prescribed. In contrast, other participants had a desire to find explanations and establish an illness notion. This was not only to fill the gaps left by healthcare providers but also to refute claims that they caused the illness themselves. For these participants, finding explanations was a way to challenge earlier misconceptions. Some emphasised that gout could affect anyone, regardless of efforts to lose weight or dietary habits. A few attributed their gout to genetic heritage—‘many relatives before me had gout’ (p 2), while others believed it was caused by specific medications—‘I know it came as a consequence of diuretics’ (p 6). Many participants acknowledged that people in general held misconceptions about gout, but they did not always feel compelled to address or correct them. They heard descriptions such as the ‘old man's illness’. One man described his feelings: ‘When one hears it, well, “port wine toe”…. I don't react to it. It's just a saying. I drink very little alcohol, so that … well, no, it doesn't affect me’ (p 8).

##### 3.1.2.2 Finding Your Own Strategies for Managing a Life With Gout

The participants expressed various approaches to managing pain and other symptoms as part of their adaptation process. These strategies reflected their ongoing struggle to cope with and adjust to physical, social and psychological levels of living with gout. For some participants, the primary strategy for managing pain was to remain inactive during gout flares. This often resulted in sedentary periods lasting up to 4 weeks: ‘All I do is sit in a chair until the pain fades’ (p 9); ‘it was hard to work sometimes’ (p 8); ‘you become completely inactive’ (p 4) and ‘you can't stand anything’ (p 10). Retired participants found it easier to accommodate the inactive periods. However, participants with young children adapted to this situation by avoiding overly strenuous activities, which led to a feeling of missing out socially with their family.

In cases where participants felt dissatisfied with the lack of effective treatment from healthcare providers, despite repeated healthcare visits, some turned to alternative medicine, such as homoeopathy. Some participants also made dietary adjustments based on experiences, identifying certain foods or drinks that seemed to trigger gout flares. One participant said: ‘It was after I had eaten pork chops or a lot of ham or offal that I felt it immediately’ (p 5).

Among participants whose symptoms were relieved through ULT, the effort to adapt to the disease was reduced. The symptom relief also reinforced their adherence to pharmacological treatment. After many years of living with gout, however, some participants still questioned whether complete remission was possible and whether they could discontinue their ULT. Nevertheless, they hesitated to discontinue ULT due to the excruciating pain they had experienced over the years. The relief provided by ULT, coupled with the absence of side effects, was a sufficient incentive for them to remain compliant and continue taking it daily.

If you take many drugs, you might consider stopping this [allopurinol]. But then, I wouldn't know how it would be if it gets worse right away. So that's why I don't dare to remove it either.

(p 1)

Neglecting the disease was another strategy employed by some participants. They mentally detached from their gout between flares and refused to let the illness dictate their lives. One participant narrated how he ignored his gout pain and continued with work‐related travel despite flares. For another participant, changing duties with a colleague made it possible to manage work despite physical limitations caused by gout: ‘I worked all the time anyway, limping. We can sort of trade jobs with each other, so I don't have to be out taking walks and so’ (p 2).

##### 3.1.2.3 Getting Used to Pain and Other Impairments

Living with gout entailed getting used to the shifting nature of pain. The gradually progressive nature of gout, with recurrent gout flares more often over time, seemed to lead to a normalisation and acceptance of pain and impairments caused by gout.

I contacted healthcare in the beginning, but thereafter, I knew why I had symptoms…. I used pain‐relieving drugs for a week or so, and it got better. However, the tendency was that the flares appeared more often.

(p 8)

Enduring pain and physical impairment were generally how living with gout was described. One man stated: ‘Yes, it hurts, but … now … after all, I can walk’ (p 3). The nature of the disease played a significant role; gout progressed gradually over many years: ‘For me, it started 35 years ago … you get used to a lot’ (p 5). This progression necessitated a level of adaptation to the symptoms. This included accepting that complete relief from pain and disabilities would never be achieved. After experiencing the initial flares, individuals learned that subsequent flares would pass, and they developed greater endurance. One man stated: ‘But then it was just a matter of enduring and staying calm, so to speak. And use pain relief. It passed, after all’ (p 5).

The experience of gout pain was described as changing in character, with flares becoming more frequent and the pain never completely disappearing between flares as time went on. Participants noted a persistent numbness and varying swelling in the affected joints during the periods between flares. One man stated, ‘it'll probably be swollen forever, my finger’ (p 10). Some participants described how the condition became more severe over time. For those who initially found relief with pain‐relieving drugs, the effectiveness of these drugs diminished over time. Others felt that the symptoms also led to physical difficulties, such as balance problems.

It's not only in the big toe, also on the footpad, it feels like walking on a pillow. You struggle with putting your toes on the ground, and it is troublesome to walk. You walk as if you were drunk…. I've had it for several years. It won't go away.

(p 3)

## 4 Discussion

This study reveals experiences of uncertainty associated with living with gout, partly explained by an unpredictability of the disease and the interactions with healthcare personnel. Below, we discuss the mismatch between participants' experiences and the support and treatment provided by healthcare, the mechanisms behind the adaptation process and the participants' lack of experience with stigmatisation.

The mismatch, as presented in our results, occurred when the participants' needs and experiences did not align with the treatment and support provided. Participants vividly described the flares as surpassing other painful experiences. The findings about the strong and often unbearable pain are consistent with several studies on the experiences of living with gout, which emphasise pain as the major problem [1, 2, 3, 31], exerting a significant impact on various aspects of life [32]. Furthermore, the mismatch, that is, the lack of alignment between patients' and healthcare personnel's views, has also been described previously when it comes to defining gout remission [33] or gout control [34]. In a study examining patients' views on how to define gout remission and establish multinational criteria [35], patients disagreed with the suggested criterion of 6 months and instead believed that remission required 12 months without a flare. The patients' definition of remission included not only fewer flares or less pain but also relief from gout‐related life impacts, such as anxiety about future flares, which took over 6 months to resolve [33]. Taken together, these studies and the findings from our study suggest that the patients' experiences of living with the disease and the personnel's starting point in treating the disease may be far apart. Bringing about positive change for patients with gout would require a shift towards making the patients' experiences the starting point for care and treatment, which is in line with the principles of person‐centred care [36]. A person‐centred approach involves viewing patients as individuals and co‐creators of their healthcare. Furthermore, it places significance on staff listening to patients' narratives in order to establish the starting point with a shared understanding of each patient's needs and wishes, thereby creating a platform for shared decision‐making about support and treatment [36].

The concept of ‘hedonic adaptation’ can be applied to the findings regarding the adaptation process in our study, providing a possible explanation for why the initial healthcare contacts seemed crucial in shaping future healthcare‐seeking behaviour. As described by Lyubomirsky [37], hedonic adaptation occurs in individuals as a response to both positive and negative experiences, where emotions that are initially intense gradually diminish as individuals become accustomed to them. This process persists as long as the situation remains relatively unchanged. However, when individuals are faced with new contexts or circumstances, a process of adapting to the new circumstances begins. In our study, the experiences of having one's symptoms neglected during the initial healthcare encounter appeared to deter participants from seeking care during subsequent flares. The avoidance of healthcare contact, leaving patients to manage their symptoms on their own, can be seen as one circumstance contributing to the adaptation process. According to Lyumbomirsky [37], well‐being or the pursuit of balance is a general goal for individuals, which includes adjusting to chronic conditions [38]. For those with rheumatic diseases, characterised by pain and disabilities, this pursuit involves striving for a life of quality while managing symptoms [39]. Hence, when participants in our study experienced that healthcare could not offer them treatment they perceived as relevant, getting accustomed to and accepting the pain caused by gout seemed necessary to restore balance in life. The diminishing negative impact of a gout flare over time could reflect this adaptation process, which may also account for patients' reduced inclination to seek care after feeling neglected during their initial contact. However, in the case of gout, adaptation to pain and disabilities can often be avoided if adequate treatment is initiated in an early phase of the gout disease [11]. Consequently, increased access to preventive treatment is imperative.

In our study, in contrast to earlier research [39, 40], participants did not emphasise the stigma surrounding gout, closely connected to lifestyle factors such as alcohol consumption and unhealthy eating habits. The closest description of stigmatisation was related to an awareness of misconceptions in society about gout being caused by excessive alcohol consumption. This shift may reflect societal progress in the destigmatisation of gout, which might differ from the context in which previous studies were conducted. However, if more participants had been included, we might have captured experiences of stigma. The experience of shame among patients seeking care has been identified as a barrier to gout care in previous studies [19, 21, 23]. According to our study, the barrier seemed to be related to a sense of resignation due to the perceived lack of adequate or individualised treatment, making patients reluctant to reach out a second time.

### 4.1 Strengths and Limitations

To create conditions for gaining a breadth of experiences, we included participants who represented a broad range of attributes as described in the introduction. However, the lack of non‐Swedish‐speaking patients is a limitation, as people from abroad make up 20% of the population in Sweden [41]. This could affect the transferability [42] of the findings to other settings and populations, despite the fact that data collection proceeded until saturation was reached. Another strength is that the first author conducted all the interviews and coordinated the data collection and simultaneous analysis in collaboration with the other authors. Moreover, the first author's nursing experience was useful when conducting the interviews, in terms of listening and paying attention to patients and their narratives. The authors encompass diverse perspectives, representing both genders, various professions (nurses, physicians and physiotherapists) and a range of clinical experiences from both internal and external viewpoints, which has characterised the analysis in a multifaceted and broad way. We intended to illuminate and increase the understanding of the long‐term experience of living with gout. However, the data was collected at a single point in time, making it challenging to recall events from years or even decades ago accurately. Nevertheless, what remains in memory may capture the core essence of their experiences. One limitation is that although the participants had been diagnosed with gout in primary care units, this was not verified for the purposes of this study. As several participants had comorbidities, they may in the interviews have described symptoms and consequences originating from conditions other than gout. Nevertheless, the most important aspect is that the data have been interpreted in the context of what participants described as their experiences and emotions of living with and receiving treatment for gout over time. To further strengthen the confirmability [43], numerous citations have been used throughout the paper.

## 5 Conclusions

This study enhances our understanding that living with gout entails significant uncertainty. This uncertainty stems from a mismatch between the care received and the individual's perception of the disease's severity and the nature of the condition, characterised by gradual progression and intermittent symptom‐free periods between gout attacks. The mismatch seemed to force an adaptation process over time, which, in turn, led some participants to refrain from seeking care when needed. The adaptation process could be significantly influenced by targeted interventions during a patient's initial healthcare encounters, such as offering information about preventive treatment, guidance on when to seek care in the future and personalised lifestyle recommendations.

## Author Contributions

**Helene Sedelius:** conceptualisation, investigation, funding acquisition, writing – original draft, methodology, validation, visualisation, writing – review and editing, formal analysis, project administration, data curation, resources. **Renée Flacking:** conceptualisation, methodology, supervision, writing – review and editing, visualisation, validation, writing – original draft, data curation, formal analysis. **Mats Dehlin:** conceptualisation, validation, visualisation, writing – review and editing, formal analysis. **Anna Svärd:** conceptualisation, funding acquisition, writing – original draft, methodology, writing – review and editing, formal analysis, project administration, supervision, data curation, resources. **Malin Tistad:** conceptualisation, writing – original draft, methodology, validation, visualisation, writing – review and editing, formal analysis, data curation, supervision.

## Ethics Statement

The study has been approved by the Swedish Ethical Review Authority (reg number 2019‐ 00077 and 2019‐04888).

## Consent

Written and oral informed consent to participate was obtained from the participants.

## Conflicts of Interest

The authors declare no conflicts of interest.

## Data Availability

The datasets generated and analysed during the current study are not publicly available because the participants did not agree to this and were assured confidentiality. Still, they are available from the corresponding author upon reasonable request.
